# Nanoseeded Desupersaturation and Dissolution Tests for Elucidating Supersaturation Maintenance in Amorphous Solid Dispersions

**DOI:** 10.3390/pharmaceutics15020450

**Published:** 2023-01-30

**Authors:** Gulenay Guner, Ayesha Amjad, Matthew Berrios, Manisha Kannan, Ecevit Bilgili

**Affiliations:** Otto H. York Department of Chemical and Materials Engineering, New Jersey Institute of Technology, Newark, NJ 07102, USA

**Keywords:** poorly soluble drugs, amorphous solid dispersions, residual crystals, nanoseeds, solvent-shift method, desupersaturation

## Abstract

The impact of residual drug crystals that are formed during the production and storage of amorphous solid dispersions (ASDs) has been studied using micron-sized seed crystals in solvent-shift (desupersaturation) and dissolution tests. This study examines the impacts of the seed size loading on the solution-mediated precipitation from griseofulvin ASDs. Nanoparticle crystals (nanoseeds) were used as a more realistic surrogate for residual crystals compared with conventional micron-sized seeds. ASDs of griseofulvin with Soluplus (Sol), Kollidon VA64 (VA64), and hydroxypropyl methyl cellulose (HPMC) were prepared by spray-drying. Nanoseeds produced by wet media milling were used in the dissolution and desupersaturation experiments. DLS, SEM, XRPD, and DSC were used for characterization. The results from the solvent-shift tests suggest that the drug nanoseeds led to a faster and higher extent of desupersaturation than the as-received micron-sized crystals and that the higher seed loading facilitated desupersaturation. Sol was the only effective nucleation inhibitor; the overall precipitation inhibition capability was ranked: Sol > HPMC > VA64. In the dissolution tests, only the Sol-based ASDs generated significant supersaturation, which decreased upon an increase in the nanoseed loading. This study has demonstrated the importance of using drug nanocrystals in lieu of conventional coarse crystals in desupersaturation and dissolution tests in ASD development.

## 1. Introduction

About 90% of newly synthesized drug molecules and 75% of drugs under development in the pharmaceutical industry are estimated to be poorly water-soluble [1,2,3]. This makes drug administration to patients a formidable challenge [4,5]. Amorphous solid dispersions, shortly ASDs, wherein a drug is molecularly dispersed in a polymer matrix, are a major formulation platform approach for enhancing the bioavailability of poorly water-soluble drugs by increasing the dissolution [6]. The high supersaturation capability of the amorphous drug and precipitation inhibition characteristics of the amphiphilic polymers enable ASDs to enhance the dissolution of poorly soluble drugs [7]. In ASDs, most of the drug is present in a molecularly dispersed, amorphous form within the ASD matrix of amorphous polymers and surfactants [6]. Compared to its crystalline form, an amorphous drug exhibits higher “kinetic solubility” and generates supersaturation during dissolution in vitro and in vivo since it has a higher free energy than its crystalline form [8]. However, this metastable nature creates a risk for precipitation or recrystallization in both solid and solution states [9,10].

Despite its beneficial aspects, amorphous drugs can precipitate during the wetting–dissolution of the ASDs, which is not favorable for their physical stability and sustained supersaturation generation capability [8,11]. High supersaturation is desirable for drug absorption and bioavailability if supersaturation is maintained during the 4–5 h gastrointestinal transit time. While this initial high supersaturation is desirable in itself, it may lead to primary nucleation and/or secondary nucleation due to residual (seed) crystals [12], followed by aggregation or growth of the precipitates and ensuing desupersaturation [4,8,11]. Depending on their concentration and properties, the polymers in ASDs can maintain supersaturation by preventing precipitation [13]. Unfortunately, these complex mechanisms during the solution-mediated precipitation of amorphous drugs, as well as the roles of residual crystals and the excipients (polymers and surfactants) in precipitation, are not yet fully understood [14,15].

Among the popular production approaches of ASDs, hot-melt extrusion (HME) may yield ASDs with residual crystals if the thermal input and mixing are insufficient so that the melting and dissolving of the drug into the molten polymer is incomplete [16,17]. On the other hand, ASDs prepared by spray-drying (SD) may contain residual crystals due to a lack of solution-state interactions between drug and polymer and/or slow drying kinetics [18,19]. It may be challenging to detect the sub-micron residual crystals using common characterization methods such as XRPD and DSC, especially at low concentrations [17,20]. Besides during the production, crystals may also form in the ASD matrix during storage when ASDs are exposed to air with high humidity and elevated temperature [21]. To evaluate the performance of the ASDs, residual crystals should be considered as they act as seeds, causing re-crystallization of the drug via nucleation and growth during storage or dissolution [22,23], jeopardizing the physical stability of the ASD. The crystal traces in ASDs also have a notable impact on drug release during the dissolution tests [24,25].

Depending on the drug–polymer miscibility and solubilization of the drug in the polymer (HME) or in the organic solvent-polymer solution (SD), which in turn depend on the processing conditions, physico-chemical properties of the drug and the polymer, as well as their composition, ASDs might have different morphologies [26]. These morphologies include (i) a single domain of homogeneously mixed drug and polymer [26,27], (ii) phase-separated domains enriched in either polymer or drug (e.g., [21,28]), and (iii) residual crystals encapsulated in the polymer matrix [17,29]. Within the context of HME, the processing conditions (temperature and screw speed) and screw configuration can be chosen in such a way as to ensure the intimate mixing of the drug particles within the polymer, down to the molecular level, for a sufficient time (residence time), which in turn can lead to ASDs with low crystallinity (or XRPD-amorphous ASD) or a fully amorphous ASDs [30]. In the melting regime of HME processing [30], the risk of residual crystallinity is almost nonexistent, whereas sufficient mixing and/or residence time must be employed to avoid the formation of residual crystals in the solubilization regime; the formation of residual crystals is unavoidable in the suspension regime [30]. While the suspension regime could lead to micron-sized coarse crystals in the ASDs, the HME process is rarely operated in this regime. Hence, with properly operated HME and SD processes, the as-produced ASDs may have phase-separated domains and nanocrystals in the sub-micron region (10–1000 nm) [17,21,26,28,29,30,31,32]. This is not surprising because, for a typical drug, the critical nucleus size is estimated to be on the order of ~10 nm; therefore, phase-separated domains or residual crystals could have <100 nm sizes [26]. On the other hand, it has been shown that upon annealing, the residual crystals in an ASD could grow up to ~30 µm [27]; upon 150-day aging of an ASD, the drug-rich phase-separated domains could grow up to several microns [33]. Obviously, these sizes depend on the specific drug–polymer formulation and processing conditions. In view of the above literature review, most ASDs, if not all, are expected to have some residual nanocrystals (10–1000 nm), which have formed during manufacturing and/or long-term storage. It is suspected that the residual nanocrystals with a larger surface area can be more detrimental than the micron-sized crystals in terms of solution-mediated precipitation and maintenance of supersaturation generated by ASDs, which is the main interest in this study.

**Figure 1:**
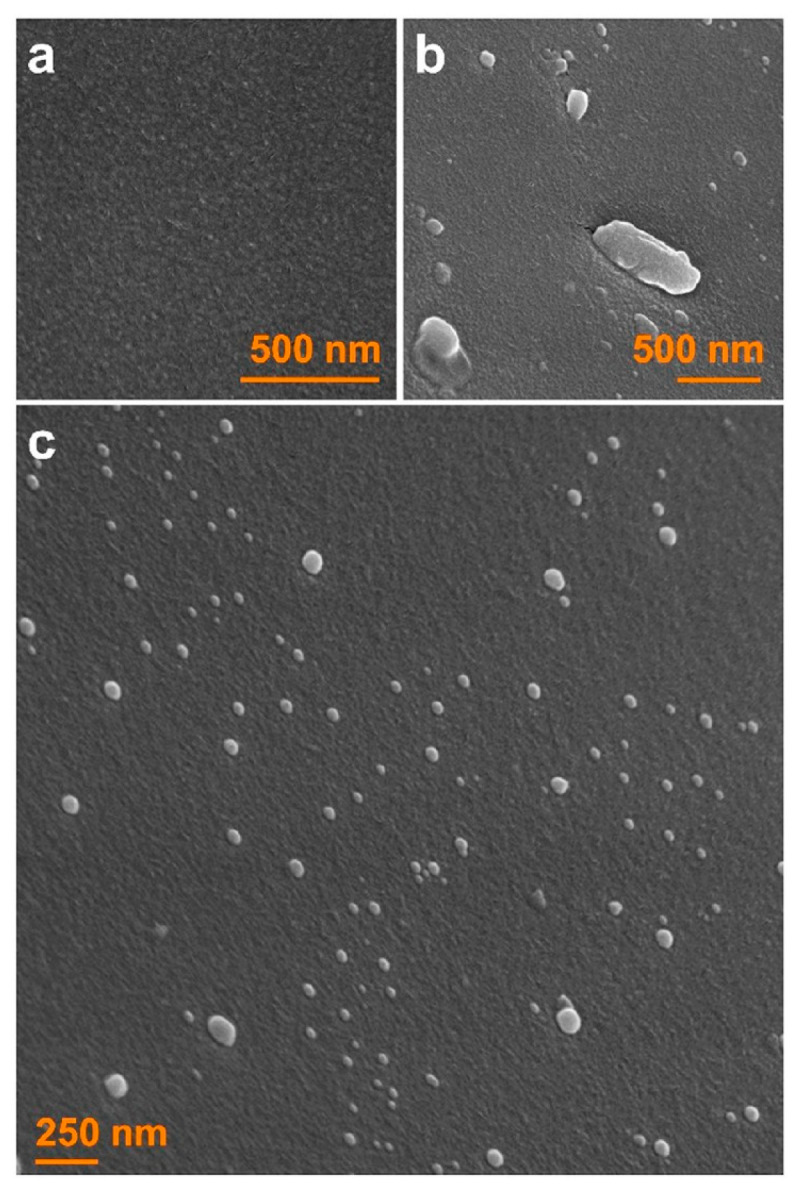
Scanning electron microscopy (SEM) images of an ASD extrudate. Some regions appear fully amorphous (**a**) or contain crystalline domains between 10 and 1000 nm (**b**,**c**). The length distribution of the domains found in (**c**) were measured as 38 ± 18 nm (±SD, n = 100). “Reprinted with permission from Moseson et al. [17]. Copyright 2022 American Chemical Society”.

Dissolution tests and solvent-shift (desupersaturation) tests are commonly used to study the impact of residual crystals. For example, ASDs that have varying levels of crystalline content (0–25%) were prepared by tuning the HME process parameters, and a reduction in the solubility advantage of the ASDs stemming from the residual crystals was demonstrated [31]. A seeded solvent-shift test was performed to decouple the supersaturation generation and precipitation–particle growth mechanisms, focusing on the latter because these mechanisms operate simultaneously in dissolution tests [34]. Seeded desupersaturation tests were performed using bulk drug microcrystals as seeds to demonstrate their facilitation of drug desupersaturation at high drug loading [31,32,35,36,37]. For most ASDs, actual residual crystals range from ten to a thousand nanometers broadly, but mostly ten to a few hundred nanometers (see Figure 1) [17,21,30,31,32]. Hence, the current testing methods cannot emulate the drug desupersaturation originating from the growth of residual “nanocrystals” because they make use of coarse micron-sized crystals [15]. However, preparing stable drug nanocrystals to be used as seeds can be challenging [38]. 

This study examines the impacts of the seed size loading on the precipitation from griseofulvin ASDs in solvent-shift (desupersaturation) tests and on drug release from ASDs during dissolution tests. A major novelty of this study is that drug nanoparticle crystals (nanoseeds) prepared by wet stirred media milling were used as a more realistic surrogate for residual crystals in ASDs and compared with the conventional micron-sized seeds of the drug. Griseofulvin was purposefully used as a model poorly water-soluble drug because it is a fast crystallizer [31] and poses serious challenges due to its fast solution-mediated precipitation [39]. ASDs with Soluplus, Kollidon VA64, and hydroxypropyl methyl cellulose at a 1:3 drug-to-polymer ratio and 0.125% (*w*/*v*) SDS were prepared by spray drying. After characterizing the seeds and spray-dried ASDs using SEM, XRPD, and DSC, the dissolution and desupersaturation profiles obtained for various loadings of conventional micron-sized seeds and nanoseeds were compared. In the solvent-shift tests, wet-milled nanoseeds were added to the GF–polymer solutions, whereas they were added to the medium during the dissolution of the ASDs. Recrystallization of GF in the presence of different crystalline seed loadings was studied for all three polymer types. The nucleation and crystal growth of GF for each polymer type were evaluated in view of the measured concentration and particle size profiles during dissolution/desupersaturation experiments. It is expected that this comprehensive analysis will allow us to elucidate the impact of seed size (surface area) and loading on the precipitation from supersaturated drug solutions. Hence, ultimately, the desupersaturation and dissolution tests with drug nanoseeds could inform the development of robust ASDs about the effectiveness of the polymers and acceptable level of residual crystals.

## 2. Materials and Methods

### 2.1. Materials

We purchased micronized griseofulvin (BP/EP grade, BCS Class II, GF) from Letco Medical (Decatur, AL, USA). The aqueous solubility of GF is 14.2 mg/L at 37 °C [40]. Its melting point *T*_m_ and glass transition temperature *T*_g_ are 220 °C and 89 °C, respectively [41]. Three polymers and one surfactant were used in the formulations. BASF (Tarrytown, NY) donated Soluplus^®^ (Sol), which is a graft copolymer made of polyvinyl caprolactam–polyvinyl acetate–polyethylene glycol, and its *T*_g_ is 73 °C [42]. Kollidon VA64 (VA64) was also donated by BASF, which is a vinylpyrrolidone-vinyl acetate copolymer with a *T*_g_ of 102 °C [43]. Dow Chemicals (Midland, MI, USA) donated hydroxypropyl methyl cellulose (HPMC, Methocel-E3 grade), which is a nonionic cellulosic polymer with a *T*_g_ of 174 °C [44]. We purchased sodium dodecyl sulfate (ACS grade, SDS) from GFS chemicals (Columbus, OH, USA), which is an anionic surfactant used to enhance the wettability of the ASD particles in the dissolution tests. We purchased acetone (ACS reagent, ≥99.5%) and ethanol (reagent alcohol, ≥95%) from BDH Analytical chemicals (Radnor, PA, USA) and used them as solvents to prepare drug–polymer solutions. We purchased the milling media, yttrium zirconia beads (Zirmil Y), from Saint Gobain ZirPro (Mountainside, NJ, USA), which had a median size of 430 μm.

### 2.2. Methods

#### 2.2.1. Nanoseed Preparation via Wet Stirred Media Milling

Before each milling experiment, a pre-suspension was prepared by dispersing 2.5% (*w*/*v*) as-received GF in 240 mL of DI water, 7.5% polymer, and 0.125% SDS under a DLM 1638 shear mixer (Cat# 14-503, Fischer Scientific, Pittsburg, PA, USA), which was operated at 300 rpm for 120 min. This formulation was selected so that the percentages of drug-polymer-surfactant would match the percentages used in the ASD formulations. In addition, this formulation was shown to yield stable nanosuspensions [45], which is desired for providing consistent seed sizes for the dissolution/desupersaturation experiments. After overnight storage at 8 °C in a refrigerator, the pre-suspensions were milled using a Microcer wet stirred media mill purchased from Netzsch, LLC (Exton, PA, USA). The milling conditions were adapted from our prior work on wet stirred media milling [39]. In total, 50 mL of the 80 mL milling chamber was filled with zirconia beads, and the beads were held inside with the help of a screen with a 200 μm opening placed at the chamber outlet. Recirculation between the mill chamber and the holding tank was provided with a peristaltic pump purchased from Cole-Palmer (Vermont Hills, IL, USA), with a flow rate of 126 mL/min. The suspension was milled for 64 min while the milling speed was set at 3200 rpm. A chiller (Model M1-0.25A-11HFX, Advantage Engineering, Greenwood, IN, USA) was used to cool the mill and the suspension to ensure its temperature was below 35 °C. Nanosuspensions were kept at 8 °C in a refrigerator. As the formulations of the nanoseeds and the ASDs had a 1:3 drug:polymer ratio, and they all contained the same small content of SDS (refer to Table 1), the formulations were labeled with Seed-polymer and Sol-polymer, respectively. The pre-suspension with Sol was also used without milling, referred to as the as-received seed (AR) in the dissolution and desupersaturation experiments, to examine the size effect of the seeds.

#### 2.2.2. Spray Drying

According to the formulations in Table 1, solution-based feeds were prepared by dissolving the drug, polymer, and surfactant in the respective solvent mixture for each polymer. Acetone was used to dissolve GF, Sol, and HPMC, whereas DI water was used to dissolve SDS. Since VA64 is not soluble in either of the solvents, ethanol was used additionally. We sonicated the solutions for 30 min prior to spray drying. A spray dryer purchased from Procept (4M8-Trix, Zelzate, Belgium) with a bi-fluid nozzle was used to dry the drug solution. The drying air temperature was set to 75 °C with a flow rate of 0.27–0.30 m^3^/min. Parameters regarding the atomization of the feed were adapted from Rahman et al. [39]. Before their characterization, we stored the spray-dried particles inside double-plastic bags placed in a vacuum desiccator.

#### 2.2.3. Particle Size and Morphology

The Rodos/Helos laser diffraction system (Sympatec, NJ, USA) was used to measure the particle sizes of the spray-dried ASDs based on Fraunhofer theory. We placed about 1 g of the powder on top of the sample chute of the dispersing system, which was vibrated at a 100% setting, and a 0.1 bar dispersion pressure sucked in the falling powder through the sample cell of the laser diffraction system. This method was adopted from ref. [46].

During seeded dissolution and desupersaturation experiments (Section 2.2.5), samples were taken at certain time points, and their particle sizes were measured using dynamic light scattering (DLS) with a Delsa Nano C Particle analyzer purchased from Beckman Coulter (Brea, CA, USA). The sizes of the seeds were also determined by DLS. The cumulant size (z-average) for all samples and the polydispersity index (PDI) of the initial and final precipitates were reported. In the seedless desupersaturation tests, only the particle sizes of the final samples were measured. In separate experiments, to investigate the presence of micelles in the desupersaturation medium, samples were taken after 3.5 h of polymer and acetone (without any drug) addition, and the sizes of the micelles (if any) were measured.

To visualize the morphology of the milled particles and confirm the particle size data obtained from DLS and Rodos/Helos qualitatively, the JEOL JSM 7900F field emission scanning electron microscope (SEM) (JEOL USA, Inc., Peabody, MA, USA) was run at 2 kV. About 0.1 mL of the Sol-based milled suspension was mixed with 10 mL deionized water in a test tube and centrifuged at 3200 rpm, as described fully in ref. [47], to remove the excess polymer. This was repeated two more times. After each centrifugation, about 8 mL of the supernatant was decanted and replaced with deionized water, partially resuspending the dense sludge at the bottom of the sample tube. Following the third centrifugation step, the addition of 8 mL of deionized water to the dense sludge led to a diluted suspension from which a droplet was taken and put on top of a carbon specimen holder and dried in a desiccator overnight. Sol-based spray-dried ASD was also placed on a carbon specimen holder. We sputter-coated all samples with gold by using BAL-TEC MED020 (BAL-TEC, Balzers, Switzerland) to decrease possible charging during imaging.

#### 2.2.4. Solid-State Characterization

The as-received drug, Sol, VA64, HPMC, physical mixtures (PMs), overnight dried seeds, and spray-dried powders were characterized using XRPD and DSC for crystallinity. An XRPD (PANanalytical, Westborough, MA, USA), equipped with Cu Kα radiation (λ = 1.5406 Å), was used to scan samples within the range of 5° to 40° at a rate of 0.165 s^−1^ in the 2θ scanning mode. A Mettler-Toledo polymer analyzer DSC (Model: DSC 3) (Columbus, OH, USA) was used by placing 6–7 mg of the samples in a sealed perforated aluminum pan of 40 μL. Spray-dried ASDs were first heated from 25 °C to 70 °C, and the temperature was kept at 70 °C for 2 min to remove any residual moisture/solvent. Then, it was cooled back to 25 °C. Finally, the samples were heated again at a rate of 10 °C/min from 25 °C to 250 °C, which was an adapted method from ref. [13]. All other samples were heated from 25 °C to 250 °C at a rate of 10 °C/min. We used nitrogen at a 60 mL/min flow rate and analyzed data using the STARe V16.20 software provided by Mettler-Toledo.

Thermogravimetric analysis (TGA) (TGA/DSC1/SF Stare system, Mettler-Toledo, Inc., Columbus, OH) was used to determine the residual solvent/moisture content in spray-dried samples. We placed 6.0–7.0 mg samples in a ceramic crucible and heated at a rate of 10 °C/min under a 60 mL/min nitrogen flow from 25 °C to 150 °C, which was adapted from ref. [13].

#### 2.2.5. Seeded Desupersaturation and Dissolution Experiments

The seeded desupersaturation and dissolution experiments were performed at varying seed percentages shown in Table 2. A Distek 2100C USP II dissolution tester (North Brunswick, NJ, USA) was used for both tests. A desupersaturation (solvent-shift) method was used to test (i) the nucleation inhibition ability of the polymers in the absence of any seeds (seedless tests) and (ii) growth/secondary nucleation inhibition capability in the presence of the seeds with varying percentages (seeded tests).

In both types of desupersaturation tests, crystal growth plays a major role; in the case of seeded tests, it is the dominant mechanism. First, 100 mg of as-received GF was dissolved in 20 mL acetone. The dissolved GF amount was kept constant regardless of the seed concentration at 100 mg to maintain similar supersaturation levels in the desupersaturation tests. This solution was subsequently added to a 1000 mL aq. solution of pre-dissolved Sol–SDS/VA64–SDS/HPMC–SDS at 37 °C that was stirred with a paddle at 50 rpm. The amount of pre-dissolved polymer and surfactant was varied according to the seed concentration used so that a 1:3 drug:polymer mass ratio was achieved in the final desupersaturation sample, including the polymers and surfactants in the seed suspension. The seed percentages in Table 2 correspond to the percentage of the weight of the drug in the seed nanosuspension with respect to the weight of the dissolved drug in the acetone solution (100 mg). The seeds were added at 1 min when sufficiently high supersaturation was achieved based on prior work [39] and exploratory experiments. At 1, 2, 5, 10, 20, 30, 60, 120, 180, and 210 min, 6 mL aliquots were taken out manually, and 3 mL of the aliquots were used for particle size measurement via DLS, as described in Section 2.2.3. The rest of the aliquots were filtered through a 0.1 μm syringe filter before UV-spectroscopy measurements. The filtered samples were diluted with the dissolution medium at a ratio of 1:5 before UV measurement. The dissolved amount of GF was measured by UV spectroscopy (Agilent, Santa Clara, CA, USA) at 296 nm. All experiments were performed thrice, and the mean and the standard deviation are shown in the figures.

Prior to the dissolution tests, an assay test was performed to assess the actual drug content in the spray-dried ASDs. In the first step of the assay test, 50 mg of the powders were dissolved in 20 mL of methanol, sonicated for 30 min, and stored overnight to ensure complete dissolution. In the second step, a 100 μL aliquot was taken and diluted with 9.9 mL methanol. The absorbances of the final solutions were measured with UV at 292 nm. Each formulation was tested 6 times, and the mean drug content along with the relative standard deviation (RSD) were reported.

In the dissolution tests, spray-dried powder samples containing 100 mg GF were weighed and added to the 1000 mL deionized water at 37 °C and stirred at a paddle speed of 50 rpm. The content of amorphous drug originating from the ASDs was kept fixed in all experiments regardless of the seed concentration to target the same supersaturation levels prior to the addition of the seeds. Seeds were added at about 10 min to ensure that sufficiently high supersaturation was already achieved for the given ASD formulation, based on exploratory experiments. The seed percentages in Table 2 correspond to the percentage of the weight of the drug in the seed nanosuspension with respect to the weight of the drug in the spray-dried ASD (100 mg). Six-milliliter samples were taken out manually at 1, 2, 5, 10, 20, 30, 60, 120,180, and 210 min. Similar to the desupersaturation test, 3 mL of the samples were used for particle size measurements, and the rest were filtered through a 0.1 μm syringe filter before UV-spectroscopy measurements. The filtered samples were diluted with 37 °C deionized water at a ratio of 1:5 before UV measurement. The dissolved GF amount was measured by UV spectroscopy at 296 nm. All experiments were performed thrice, and the mean and standard deviation are shown in the figures.

## 3. Results

### 3.1. Properties of the Seeds and Spray-Dried Powders

**Figure 2:**
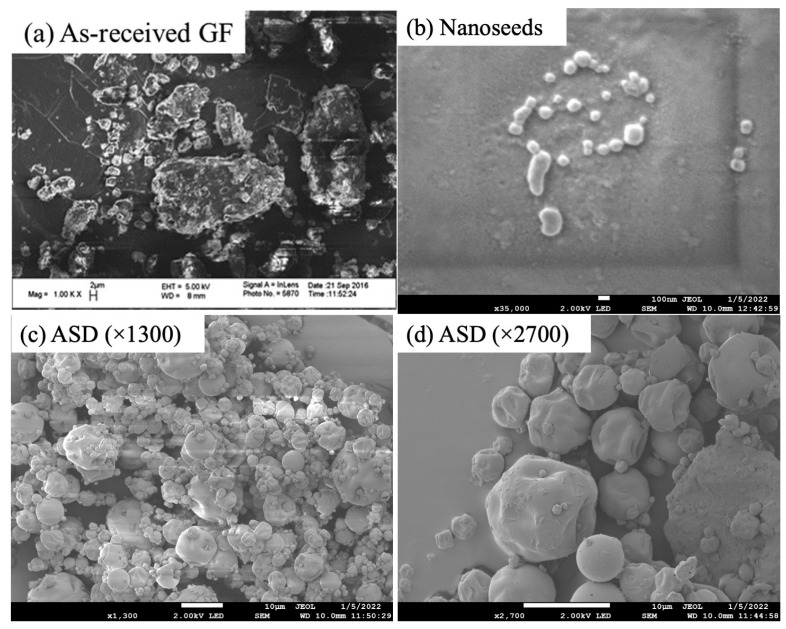
SEM images of (**a**) as-received GF as well as Soluplus-based (**b**) nanoseeds (×30,000 magnification, scale bar: 100 nm), (**c**) spray-dried ASD (×1300 magnification, scale bar: 10 µm), and (**d**) spray-dried ASD (×2700 magnification, scale bar: 10 µm).

Nanoseeds were prepared successfully via wet stirred media milling. The median particle size *d*_50_ of the as-received GF was 13.7 µm, and the milled particles had a median size in the range of 146–223 nm, depending on the formulation. Their sizes were also measured before each seeded desupersaturation/dissolution experiment, and no noticeable deviation was observed. Spray drying was performed to prepare GF ASDs. The TGA results indicated ~2.0% weight loss, which refers to any residual solvent/moisture from spray drying and moisture sorbed from the air during the processing, sampling, and measurements (room at 23 °C, ~40% RH) (e.g., [21]). In view of the higher normal boiling point of water (100 °C) as compared with those of acetone and ethanol (56 °C [48] and 78 °C [49], respectively), it is reasonable to expect that the residual liquid in the ASDs was significantly enriched in water because low-boiling solvents are preferentially removed during the spray-drying, leading to negligible organic solvent amounts in the ASDs (e.g., [50]). Table 3 presents the characteristic particle sizes *d*_10_, *d*_50_, and *d*_90_ of the ASDs, where the median size ranged from 5.15 to 8.82 µm. The particle sizes observed through SEM images corroborate the particle size measurements by DLS and laser diffraction. Figure 2a shows the large as-received GF particles with irregular shapes, while Figure 2b shows individually standing, non-aggregated rounded GF particles whose sizes match the DLS measurements qualitatively. Figure 2c,d depicts that the spray-dried ASD had spherical particles whose sizes fall within the particle size distribution measured by laser diffraction.

**Figure 3:**
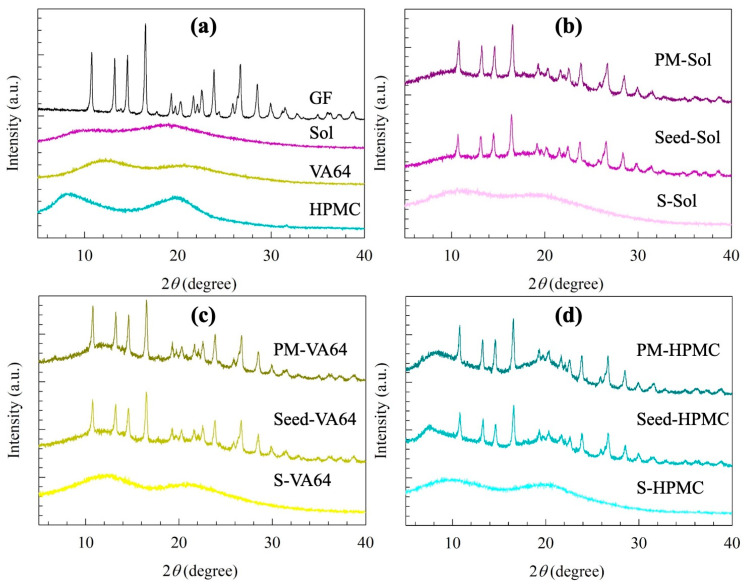
XRPD diffractograms of (**a**) as-received GF, Sol, VA64, and HPMC as well as of physical mixtures (PMs) of GF-polymer-SDS, overnight-dried nanoseeds, and the spray-dried ASDs containing (**b**) Sol, (**c**) VA64, and (**d**) HPMC as the polymer.

**Figure 4:**
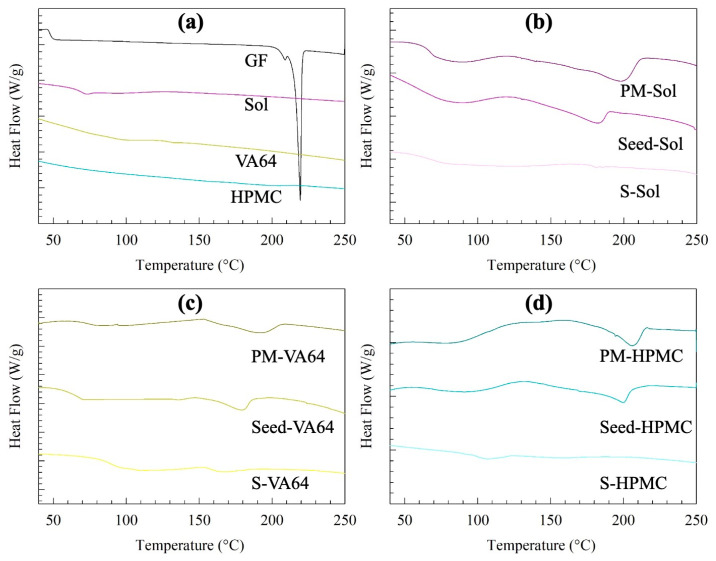
DSC thermograms of (**a**) as-received GF, Sol, VA64, and HPMC as well as of physical mixtures (PMs) of GF-polymer-SDS, overnight-dried nanoseeds, and the spray-dried ASDs containing (**b**) Sol, (**c**) VA64, and (**d**) HPMC as the polymer.

The solid state of the seeds and spray-dried ASDs was examined with XRPD and DSC, as can be seen in Figure 3 and Figure 4**,** respectively. The XRPD diffractograms of GF, the PMs, and the seeds exhibited characteristic high-intensity diffraction peaks of GF at 13.2°, 14.6°, and 16.5°, which are in accordance with the peaks previously reported for GF [39]. The diffraction peaks of GF were superimposed on the halo-patterned background of the amorphous polymer. The PMs and seeds exhibited peaks at the same diffraction angles as the as-received GF with reduced peak intensity. This reduction can be attributed to the high dilution and surface coverage with the excipients [13,51]. Moreover, the peaks for the seeds were slightly shorter compared to the physical mixture, which can be explained by better coverage of drug particles by the polymer and/or defects that formed during the milling [52]. All spray-dried powders had halo-patterned XRPD diffractograms, confirming GF ASD formation.

DSC traces corroborate the observations from XRPD diffractograms. The PMs and the seeds exhibited a lower melting point temperature and fusion enthalpy than those of the as-received GF. The seeds containing nanomilled GF had lower values than those of the PMs (see Table S1, Supplementary Materials for the actual values). These findings can be attributed to the polymer–drug miscibility [37] as well as the presence of nanocrystals and small defects, which were generated during the milling, in the seeds vs. coarse micro-sized crystals in the PM. On the other hand, the ASDs did not experience any melting event; they had a single glass transition temperature, which also indicates the molecular miscibility of GF with the polymers.

### 3.2. Solution-Mediated Precipitation of GF in the Solvent-Shift and Dissolution Tests

**Figure 5:**
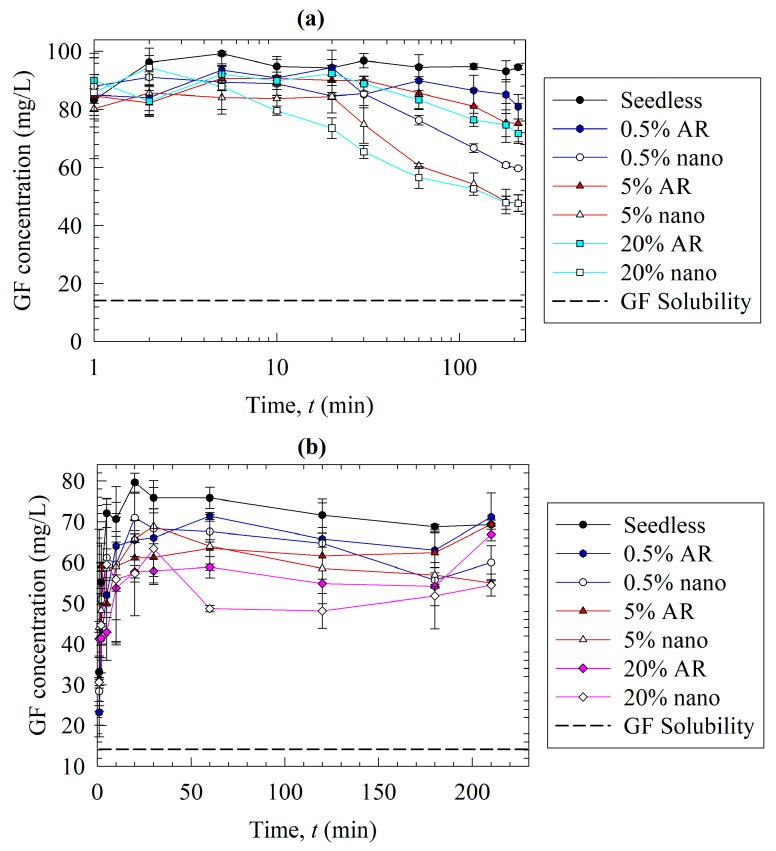
Effects of seed loading and size on (**a**) the desupersaturation in the solvent-shift test and (**b**) drug release from the S-Sol ASD in the dissolution test. In the solvent-shift tests, a 20 mL GF–acetone solution was mixed with 1000 mL aqueous solutions of Soluplus and SDS to generate supersaturation. Nanoseeds (nano) and micron-sized as received (AR) seeds were added at the weight percentages reported in Table 2.

The temporal evolution of the GF concentration during the solvent-shift (Figure 5a) and the dissolution tests (Figure 5b) were measured for various loadings of the nanoseeds and as-received (AR) seeds of GF. Note that GF nanoseeds (nano) with the Sol-based formulation prepared by wet media milling and the as-received (AR) GF seeds were added in the form of a suspension to the respective media. The AR refers to an unmilled pre-suspension consisting of micron-sized GF crystals with *d*_50_ = 13.7 µm in an aqueous Sol–SDS solution. Prior investigations [31,32] made use of similar micron-sized bulk crystals, unlike the nanoseeds, in solution-mediated precipitation studies.

In the solvent-shift (desupersaturation) tests (Figure 5a), GF attained a max. concentration (*C*) of ~90–100 mg/L, above the GF solubility (*C*_eq_), within a few minutes due to the fast mixing of the solvent stream (GF in acetone) with the antisolvent (aq. solution of Sol–SDS). The zig-zags in the timewise variation of the concentration originated from the imperfect mixing in the vessel, resulting in spatial inhomogeneity of the GF concentration, as well as the stochastic nature of the primary and/or secondary nucleation. The stirrer speed affects the hydrodynamics in the vessel, mixing degree, and solute mass transfer to the seed surfaces. Hence, it may be optimized to reduce these variations. In the absence of the seeds (seedless test), the high supersaturation attained was maintained approximately for 210 min by Sol acting as a nucleation inhibitor. Mahbub et al. [13] found that GF concentration decreased from ~98 mg/L to 35 mg/L within 24 min and to 24 mg/L at 210 min in the absence of any nucleation inhibitors, which is in line with the fast-crystallizing nature of GF [53]. When the seeds were present, however, a significant reduction in the GF concentration and supersaturation occurred due to the growth of the added seeds and/or secondary nucleation. A higher seed loading and smaller seed size led to faster desupersaturation. The difference in the desupersaturation behavior with different seed sizes and loading can be explained by the following equation:

(1) $$\frac{dm}{dt}=R_{G}=Ak_{G}S^{g}$$

where the mass (*m*) deposited on the crystal per unit time (*t*), i.e., crystal growth rate (*R*_G_), is a function of the surface area of the crystals (*A*), the crystal growth rate constant (*k*_G_), and the supersaturation (*S = C – C*_eq_) to the power of the overall growth order (*g*) [15,54]. As the experiments were performed with the same formulation and procedure, any difference in the crystal growth rates is expected to mainly originate from the surface area of the seeds. The as-received seed size (13.72 µm) is 78.5-fold of the nanoseed size (175 nm). Assuming a spherical shape and nonporous crystals as a first approximation, this suggests that the nanoseeds have a 78.5-fold higher external surface area than the as-received seeds. Hence, the more surface area provided by nanoseeds led to faster growth compared to the as-received seeds. Additionally, at the higher seed loading, the surface area of the seeds was higher, which could also explain the faster desupersaturation observed in Figure 5a.

In the dissolution tests (Figure 5b), as the GF had to dissolve and was released from the ASD particles, the supersaturation build-up was slower, and it did not reach the 90–100 mg/L level observed in the solvent-shift test. Clearly, there exists a competition between drug release from the ASD and the precipitation of the dissolved drug during the dissolution of the ASDs (also see ref [31]). Aside from this initial phase of supersaturation build-up, the impact of the presence of seeds and their concentration was similar for both tests. As the seed loading increased, the desupersaturation due to seed growth and possible secondary nucleation occurred faster, with either nanoseeds or the AR seeds. When the profiles at the same seed loading in Figure 5a were compared for different seed sizes, we found that the nanoseeds led to faster desupersaturation compared to the as-received seeds in the desupersaturation test. A similar observation can be made from the dissolution tests (Figure 5b); however, the dissolution profiles of the nanoseeds and the as-received seeds were closer to each other as compared with the respective profiles in the solvent-shift tests. This could be partly explained by the incomplete dissolution of the drug due to the aforementioned competition. Our findings overall suggest that there is a notable difference in the solution-mediated recrystallization of GF when nanoseeds vs. as-received bulk crystals are used as surrogates for the actual residual crystals. This is not very surprising because the nanoseeds used in this study (refer to Figure 2b) could better represent the actual residual nanocrystals (e.g., Figure 1b,c) than the as-received micron-sized seeds (see Figure 2a). This effect should not be neglected as it may cause misleading conclusions about the polymers’ secondary nucleation and growth inhibition abilities in the presence of residual crystallinity. Therefore, the rest of the analysis was performed by using nanoseeds only.

### 3.3. Roles of Polymers on Recrystallization Inhibition in the Presence of Nanoseeds

**Figure 6:**
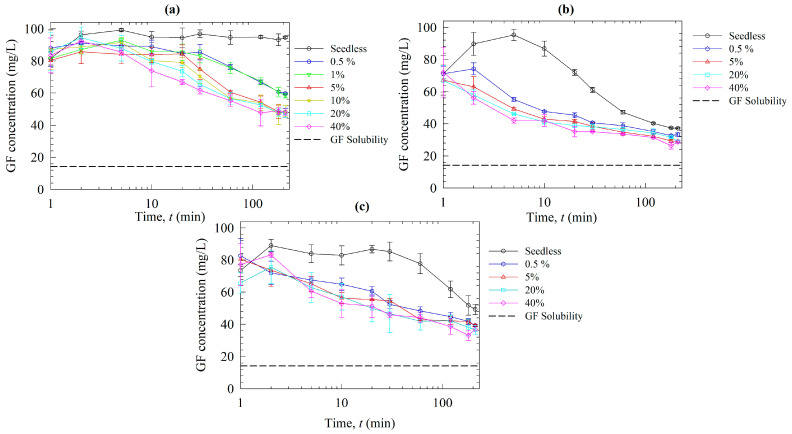
Effects of seed loading on the desupersaturation in the solvent-shift test when a 20 mL GF–acetone solution was mixed with 1000 mL aqueous solutions of SDS and various polymers: (**a**) Sol, (**b**) VA64, and (**c**) HPMC. Nanoseeds were added to the supersaturated solution at the weight percentages reported in Table 2.

Figure 6 shows the time-wise evolution of GF desupersaturation in the presence of various polymers without (seedless) and with the nanoseeds. A logarithmic time scale was used in Figure 6 for clarity of data at the earlier time points that correspond to supersaturation build-up, and the same data are presented in Figure S1 with a linear time scale. First, let us examine the seedless desupersaturation test results, which shed light on the possible primary nucleation inhibition capability of the respective polymers. Only Sol was able to prevent primary nucleation and maintain supersaturation for 210 min, whereas HPMC and VA64 were not capable. On the other hand, HPMC slowed down the crystal growth more effectively than VA64. Clearly, they were not effective nucleation inhibitors. These findings agree with those in other studies in the literature [13,55].

Next, let us examine the seeded tests. Interestingly, Sol could not completely prevent crystal growth in the presence of even 0.5% nanoseeds. While being an excellent nucleation inhibitor of GF, Sol appears not to be an excellent growth inhibitor of GF. The high surface area of the nanoseeds, coupled with the inherent fast-crystallizing nature of the GF [53], could explain these findings. At the low nanoseed loading (0.5, 1%), the desupersaturation was relatively slow, and the GF concentration was above ~60 mg/L, whereas it was much faster at the higher nanoseed loading (5–40%). For HPMC and VA64, the impact of seed loading was not as distinct as that for Sol between the low and high seed loading. The mere presence of 0.5% nanoseeds led to desupersaturation down to 40 mg/L within 52 and 196 min for VA64 and HPMC, respectively.

A characteristic kinetic behavior of all desupersaturation profiles in Figure 6 and Figure S1 is that after the initial supersaturation build-up upon solvent shift, the GF concentration, also supersaturation, dropped somewhat monotonically in time and approached an apparent plateau concentration at 210 min in some cases. It is well-known that nucleation and crystal growth are driven by the natural logarithm of the supersaturation ratio *S*_r_ *= C/C*_eq_ and the power of *S* = *C* – *C*_eq_, respectively [12,15,54], the latter of which can be seen from Equation (1). In view of this fact, we note that the driving force for nucleation and crystal growth dramatically decreased during the test, which in turn explains the continuously decreasing slope of the concentration profiles (see Figure S1 with identical data to Figure 6, but with the linear time scale). Note that in a seedless desupersaturation test conducted by Rahman et al. [13], the concentration of a supersaturated GF solution without any inhibitors was 24 mg/L at 210 min, which was still above the equilibrium GF concentration of 14.2 mg/L at 37 °C. This demonstrates that prolonged times would be needed for complete desupersaturation to attain saturation solubility of the GF crystals, which was also observed in ref. [35]. It is most likely that an apparent plateau concentration was approached at ~210 min because of the low supersaturation and the inhibiting action of the polymer. The GF concentration at 210 min ranged from 45–60, 28–34, and 36–39 mg/L for Sol, VA64, and HPMC, respectively, for the various seed loadings examined. These findings overall suggest the following rank-ordering for the overall effectiveness of the polymers in terms of precipitation inhibition: Sol > HPMC > VA64 >> No inhibitor.

**Figure 7:**
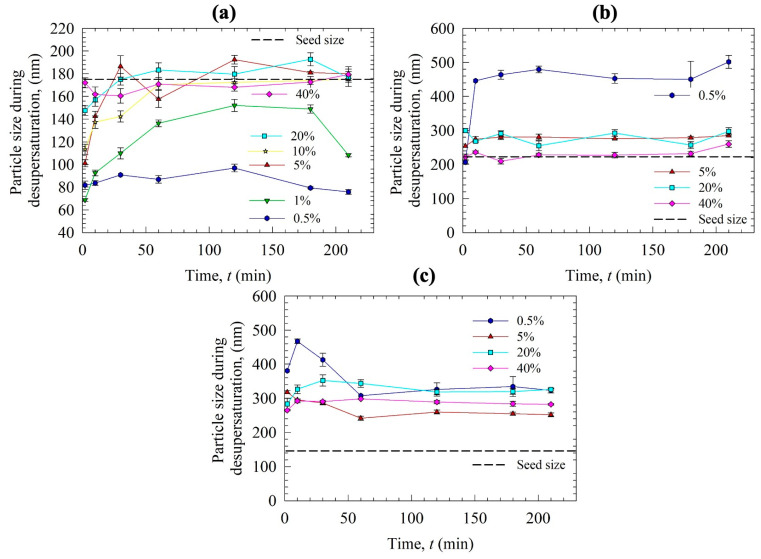
Particle size variation during the seeded desupersaturation when a 20 mL GF-acetone solution was mixed with 1000 mL aqueous solutions of SDS and various polymers: (**a**) Sol, (**b**) VA64, and (**c**) HPMC. Nanoseeds were added to the supersaturated solution at the concentrations reported in Table 2.

To gain further insights into the nucleation/growth mechanisms, particle sizes in the desupersaturation medium during the experiments were measured offline using DLS. Figure 7 presents the timewise evolution of the cumulant particle size during the desupersaturation, while the initial and final cumulant sizes and PDIs are reported in Table S2 (Supplementary Materials). As can be seen from Figure 7a, interestingly, the particle size in the medium was initially lower than the nanoseed size, and it increased gradually to a plateau size in the case of Sol as the GF supersaturation depleted in time. We hypothesize that there exist two different populations in the medium: GF nanoseeds that grew in time and Sol micelles, which could affect the overall particle size distribution notably due to the 1:3 GF:Sol mass ratio. In fact, we attribute the initial small sizes measured by DLS to the presence of sub-100 nm Sol micelles, as Sol is known to form micelles [56,57]. Rahman et al. measured the micelle sizes during the dissolution of GF–Sol ASD, where the Sol concentration was the same as in this study, by DLS, and reported the cumulant size as 80 nm [13]. In the presence of the solvent (acetone), 60 nm micelles for Sol were measured, whereas micelles were not detected for VA64 and HPMC. Hence, the sizes below the nanoseed size in Figure 7a are most likely due to the presence of the Sol micelles. Assuming the Sol micelle size did not change for different seed loading, we attribute the gradual increase in particle size to nanoseed growth in the presence of Sol micelles. Similar to Figure 6a, a differentiation can be made for the low seed loading (0.5 and 1%) vs. the high seed loading (>5%). Due to the spatial inhomogeneity of local supersaturation and potential sampling errors, there were some fluctuations superimposed on the general trend when seed loading was at and above 5%. This could also be related to possible secondary nucleation after the addition of the seeds and the stochastic nature of nucleation. Smaller particles were detected for the low seed loading, which corresponds to the lower desupersaturation in Figure 6a. Due to the complexity of the growing drug nanoseeds-Sol micelles, we can only speculate about the decrease in particle size for the low nanoseed loading (0.5 and 1%) after 120 min and attribute it to some micellar dissolution of GF of the nanoseed particles.

For VA64 and HPMC experiments (Figure 7b,c), the particles were larger than the nanoseeds from the start, and the growth trend was not clear. No micelles of HPMC and VA64 were detected when HPMC and VA64 were dissolved in the DI water–acetone mixture that had the same ratio as in the desupersaturation tests. The resultant particles were much bigger than the initial nanoseeds; the particles were also much larger than those measured in the presence of Sol. Moreover, the growth appears to be much faster when HPMC and VA64 were used, which is in line with Figure 6b,c. As the samples taken from the vessel were tested for size within the subsequent few minutes by DLS, the initial nanoseeds already grew fast. Hence, considering these limitations of the offline DLS measurements, all size measurements in this study are meant for a qualitative comparison that could help to rank-order the inhibition impact of different polymers as an orthogonal characterization tool to concentration measurement, despite being less accurate and precise.

During the seedless desupersaturation tests, particle size measurements were performed on the samples taken at the end (210 min), and the hemispherical bottom section of the dissolution vessel was observed for any precipitates. The sample with Sol had a 70 nm size, which was slightly bigger than the micelle size (60 nm, which is in line with the slight desupersaturation in Figure 6a). Our aim was to detect the size of the precipitates when VA64 and HPMC were used as the inhibitors; however, the size measurement indicated zero particle size, probably due to the very low concentration of the precipitates in the samples. The visual observation of the vessel suggests that the vessel did not have any settled coarse precipitates in the case of Sol. On the other hand, the vessel had precipitates spread at the hemispherical bottom section, which could be clearly seen for VA64, whereas only a few bigger precipitates settled at the bottom and could not be as clearly seen for HPMC. Since the precipitates were settling at the bottom, their sizes could not be measured in the DLS.

**Figure 8:**
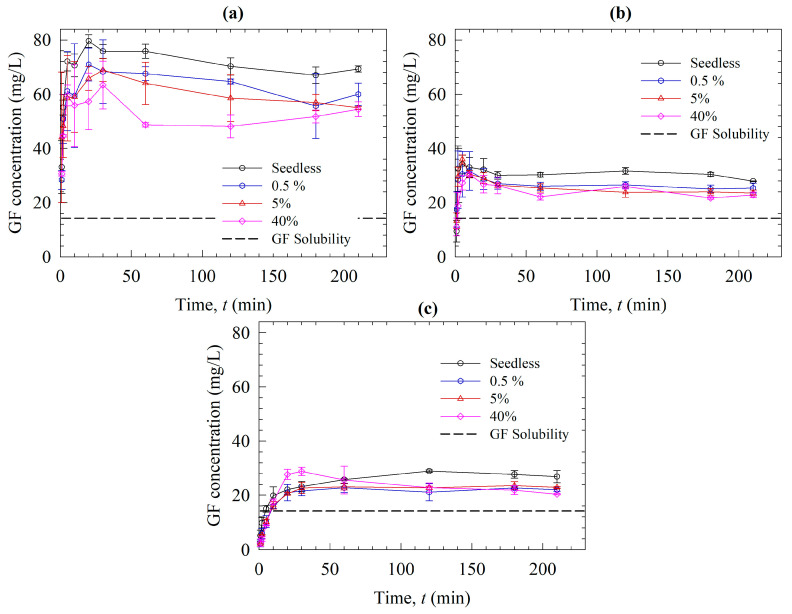
Evolution of the drug release from the spray-dried ASDs with various polymers: (**a**) Sol, (**b**) VA64, and (**c**) HPMC, in the absence of seeds (seedless) and when various percentages of the nanoseeds were added in the dissolution test.

Let us now examine the dissolution profiles of the ASDs (Figure 8): Sol outperformed VA64 and HPMC by generating higher GF supersaturation. This is due to the better precipitation inhibition capability of Sol compared to the other polymers, as seen in Figure 6. Additionally, higher solubilization of GF in Sol micelles may contribute to this outcome [45]. Sol is also known to be a better matrix recrystallization inhibitor of GF than VA64 and HPC. This phenomenon was tested under polarized light microscopy by Rahman et al. [13]. In that investigation, a loose compact of ASD powders was put in a microscopic slide, and a droplet of deionized water was poured, which caused recrystallization of the ASD with the cellulosic polymer in 1 min and VA64 in 10 min, whereas Sol remained amorphous at the end of 20 min (refer to Figure S1 in Supplementary Materials). These results could shed light on why and how Sol-based ASDs could provide significant supersaturation, whereas the ASDs with the two other polymers could not provide significant supersaturation.

**Figure 9:**
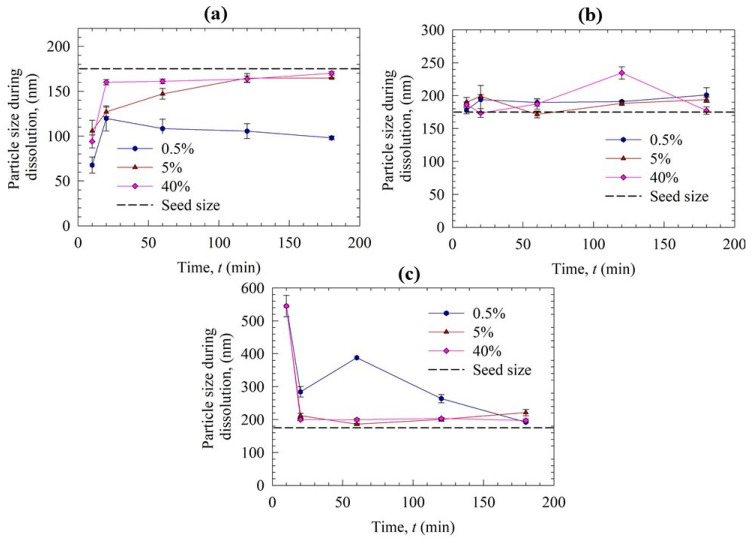
Particle size variation during the dissolution of the ASDs with various polymers: (**a**) Sol, (**b**) VA64, and (**c**) HPMC when various percentages of the nanoseeds were added in the dissolution test.

The seeds were added at 10 min of the dissolution experiments to investigate their impact when the dissolution medium had high supersaturation. Like the seeded desupersaturation experiments, the presence of the seeds caused desupersaturation via seed growth and potential secondary nucleation, which was enhanced at higher nanoseed loadings. None of the polymers could maintain a high supersaturation of fast-crystallizing GF in the presence of the nanoseeds, even though the supersaturation level was not as high as that in the desupersaturation experiments. Similar to the particle sizes in the desupersaturation tests, in the case of Sol, particles in the dissolution medium were smaller than the GF nanoseeds due to the presence of the Sol micelles. In the absence of any micelles for the VA64 and HPMC cases, the particles were much bigger than those in the Sol case. When Figure 7 and Figure 9 are compared, it is noted that the particle sizes during the dissolution tests were smaller than those in the desupersaturation tests. Note that both tests were conducted in the same vessel with identical formulation/GF nanoseed formulations. This difference could be explained by the fact that higher supersaturation levels were achieved in the desupersaturation tests (Figure 6) than in the dissolution tests (Figure 8). As explained earlier, this occurrence stemmed from the competition between supersaturation generation and recrystallization/crystal growth, as well as the matrix recrystallization observed in VA64- and HPMC-based ASDs. The supersaturation is the driving force for nucleation and crystal/seed growth (refer to Equation (1)).

Overall, Sol appears to be a better inhibitor of GF recrystallization than VA64 and HPMC, and it is an excellent nucleation inhibitor, as deduced from the seedless desupersaturation tests. This can be explained by kinetic and thermodynamic considerations, respectively, as follows: (i) Sol provided a lower driving force for drug nucleation–crystal growth thanks to the solubilization of GF molecules in the Sol micelles [13], which diminishes the concentration of the free GF molecules available for nucleation–growth and the “true supersaturation”, and (ii) Sol is more hydrophobic than HPMC and VA64, which may have facilitated its adsorption onto the growing GF crystals. The adsorbed polymer layer may block the sites on the crystal surfaces during the integration of new growth units or drug molecules into the crystal surfaces [58], thus poisoning crystal surfaces and inhibiting their growth. Polymers with higher hydrophilicity have been found to have a reduced power in suppressing nucleation than those with higher hydrophobicity/amphiphilicity [59,60,61]. Intermolecular forces may be represented by the solubility parameter, which also correlates with the solubility of polymers [62]. A higher solubility parameter indicates more hydrophilicity [58]. The Hansen solubility parameter of Sol, VA64, and HPMC is 19.4 [43], 19.7 [43], and 24.4 [63] MPa^1/2^, respectively, whereas that of GF is 24.9 MPa^1/2^ [64]. First, the drug–polymer solubility parameter differences are all below 7 MPa^1/2^, signifying polymer–drug (GF) miscibility [65], which is in line with the DSC results. Second, in terms of hydrophobicity, the polymers can be ranked as follows: Sol > VA64 >> HPMC. In a separate study, the wettability enhancement of GF in deionized water in the presence of polymers was examined via a modified Washburn method [13]. Based on the increase in the ratio of cos*θ*, i.e., cosine of the wetting angle *θ*, with respect to that of water, the hydrophilicity of the polymers was rank-ordered as HPC > VA64 >> Sol. Hence, overall, one can conclude Sol is the most hydrophobic and HPMC is the most hydrophilic among the polymers studied. While the lowest hydrophilicity of Sol could explain its excellent nucleation inhibition capability in view of [59,60,61], the hydrophobicity does not appear to explain the better growth inhibition capability of HPMC as compared with VA64. The elucidation of the growth inhibition capability entails examining the surface coverage of the drug particles with the adsorbed polymer (e.g., [32]). Overall, for fast crystallizing drugs such as GF, either a more effective nucleation–growth inhibiting polymer or a combination of polymers is warranted, whose study is beyond the scope of this study.

## 4. Conclusions

This study has demonstrated that the use of as-received micron-sized crystals, as a surrogate for residual crystals in ASDs, in seeded desupersaturation and dissolution tests could lead to misleading results. The residual crystals in ASDs tend to be 10–1000 nm. Hence, drug nanoseeds, prepared by wet stirred media milling, are the natural choice for preparing seeds to emulate the behavior of residual crystals. Drug (GF) nanoseeds led to faster growth and desupersaturation owing to their larger surface than the micron-sized crystals. Moreover, the higher seed loading also led to faster desupersaturation. Both the desupersaturation tests and the dissolution tests suggest that Sol is a better nucleation inhibitor than HPMC and VA64 owing to its higher hydrophobicity; however, it was not an excellent growth inhibitor, which became notable in the presence of even 0.5% nanoseeds. The overall GF precipitation inhibition effectiveness of the polymers was ranked-ordered as Sol > HPMC > VA64. Dissolution test results were somewhat similar to the desupersaturation test results for the recrystallization inhibition properties of the polymers. However, there were also stark differences. The solvent-shift method decouples the solution-mediated recrystallization from the supersaturation generation from the ASDs, as both phenomena compete in the dissolution tests. Moreover, it is easier to fine-tune and set the supersaturation levels and elucidate the impact of different polymers on precipitation inhibition in the solvent-shift tests. Finally, a higher extent of seed growth occurred in the desupersaturation tests as compared with the dissolution tests. This could render the seeded desupersaturation tests more stringent in terms of robust ASD development. Our findings with the nanoseeds could have much wider applicability to other drug–polymer pairs. In a future study, the solution-mediated recrystallization of a slow-crystallizing drug in the presence of various polymers and nanoseeds will be examined. Moreover, the impact of the seed size loading should also be studied in a biorelevant desupersaturation and dissolution media. The impact of the stirrer speed on the desupersaturation response, both in terms of kinetics and the concentration variability in the tests, also warrants further investigation.

## Figures and Tables

**Table 1:** Formulations of the milled aq. GF suspensions that contain the nanoseeds (Seed-Polymer) and the solution-based (S-Polymer) feeds to the spray drier for the preparation of the ASDs.

Formulation	GF (% *w*/*v*) ^a^	Polymer (% *w*/*v*) ^a^	SDS (% *w*/*v*) ^a^	Water (mL)	Acetone (mL)	Ethanol (mL)
Seed-Sol	2.5	7.5	0.125	240	0	0
Seed-VA64	2.5	7.5	0.125	240	0	0
Seed-HPMC	2.5	7.5	0.125	240	0	0
S-Sol	2.5	7.5	0.125	40	200	0
S-VA64	2.5	7.5	0.125	40	140	60
S-HPMC	2.5	7.5	0.125	40	200	0

^a^ % *w*/*v* with respect to the total liquid volume (240 mL).

**Table 2:** Seed percentages used in the desupersaturation and dissolution experiments.

Polymer in the Seed	Test	Seed (% *w*/*w*)
Sol	Desupersaturation	0
	Dissolution	
	Desupersaturation	0.5
	Dissolution	
	Desupersaturation	1
	Desupersaturation	5
	Dissolution	
	Desupersaturation	10
	Desupersaturation	20
	Desupersaturation	40
	Dissolution	
VA64	Desupersaturation	0
	Dissolution	
	Desupersaturation	0.5
	Dissolution	
	Desupersaturation	5
	Dissolution	
	Desupersaturation	20
	Desupersaturation	40
	Dissolution	
HPMC	Desupersaturation	0
	Dissolution	
	Desupersaturation	0.5
	Dissolution	
	Desupersaturation	5
	Dissolution	
	Desupersaturation	20
	Desupersaturation	40
	Dissolution	

**Table 3:** Particle size statistics of the spray-dried powders and their drug content.

Formulation	Particle Size Statistics of the Spray-Dried Particles (µm)			Drug Content, Relative Standard Deviation (% *w*/*w*, %)
	*d*_10_ ± SD	*d*_50_ ± SD	*d*_90_ ± SD	
S-Sol	1.06 ± 0.0	5.15 ± 0.1	13.17 ± 0.2	22.2, 0.51
S-VA64	2.07 ± 0.0	8.81 ± 0.1	19.30 ± 0.3	20.9, 1.1
S-HPMC	1.85 ± 0.0	7.38 ± 0.2	22.57 ± 0.7	23.7, 1.4

## Data Availability

The data are contained within the article and its Supplementary Materials.

## Supplementary Materials

The following are available online at: https://www.mdpi.com/article/10.3390/pharmaceutics15020450/s1, Table S1: Characteristic temperatures-enthalpy values obtained from the DSC thermograms; Table S2: Particle size statistics of the seeds and the precipitates during dissolution and desupersaturation experiments; Figure S1: Effects of seed loading on the desupersaturation in the solvent-shift test when a 20 mL GF–acetone solution was mixed with 1000 mL aqueous solutions of SDS and various polymers: (a) Sol, (b) VA64, and (c) HPMC. Nanoseeds were added to the supersaturated solution at the weight percentages indicated; Figure S2: PLM images of a loose compact of the spray-dried ASD particles with 1:3 drug:polymer mass ratio in 40 μL deionized water: (a) S-HPC, (b) S-VA64, and (c) S-Sol, respectively. 20 μL deionized water was added initially and rest of the 20 μL water was added after 10 min. The images were taken at 0 (before adding water), 1, 5, 10, and 20 min after the addition of deionized water. Except 0 min image (5X magnification, scale bar: 200 μm), which focused on the compact, all other images focused on particles that emanated from the surface, which were captured at 20X magnification (scale bar: 50 μm).
