# Supplementary material for: Nanoseeded Desupersaturation and Dissolution Tests for Elucidating Supersaturation Maintenance in Amorphous Solid Dispersions
Source: Pharmaceutics. 2023 Jan 30;15(2):450. doi: 10.3390/pharmaceutics15020450 (PMC9964794; doi:10.3390/pharmaceutics15020450)
Supplement: Supplementary file 1 [file pharmaceutics-15-00450-s001.zip › pharmaceutics-2174645-supplementary.pdf]

pharmaceutics

MDPI

Supplementary Materials:

Nanoseeded Desupersaturation and Dissolution Tests for Elucidating Supersaturation Maintenance in Amorphous Solid Dispersions

Gulenay Guner, Ayesha Amjad, Matthew Berrios, Manisha Kannan and Ecevit Bilgili *

Otto H. York Department of Chemical and Materials Engineering, New Jersey Institute of Technology, Newark, NJ 07102, USA
* Correspondence: bilgece@njit.edu; Tel.: +1-973-596-2998; Fax: +1-973-596-8436

Table S1. Characteristic temperatures–enthalpy values obtained from the DSC thermograms.

Polymer used in the formulation[a]	Formulation type	T_g (°C)	T_m (°C)	ΔH_f (J/g)
	PM-	N/A	200	6.9
Sol	Seed-	N/A	184	5.2
	S-	80	N/A	N/A
	PM-	N/A	193	7.1
VA64	Seed-	N/A	180	6.7
	S-	98	N/A	N/A
	PM-	N/A	217	9.8
HPMC	Seed-	N/A	201	8.8
	S-	101	N/A	N/A

[a]As-received GF had a melting point temperature T_m and glass transition temperature $T_{g.}$ of 220 °C and 89 °C, respectively.

Table S2. Particle size statistics of the seeds and the precipitates in the dissolution and desupersaturation experiments.

Formulation	Test	Seed (% w/w)	Seed		Initial precipitate		Final precipitate	
			Cumulant size (nm)	PDI	Cumulant size (nm)	PDI	Cumulant size (nm)	PDI
	Desupersaturation	0.5			81.7	0.260	73.9	0.259
	Dissolution				67.7	0.105	98.0	0.249
	Desupersaturation	1			68.7	0.134	108	0.293
	Desupersaturation	5			101	0.250	180	0.246
Seed-Sol	Dissolution		175	0.116	106	0.132	165	0.212
	Desupersaturation	10			113	0.239	175	0.201
	Desupersaturation	20			147	0.217	176	0.175
	Desupersaturation	40			172	0.182	179	0.126
	Dissolution				94.0	0.140	170	0.170
	Desupersaturation	0.5			207	0.085	501	0.218
	Dissolution				178	0.126	209	0.147
	Desupersaturation	5			254	0.106	285	0.127
Seed-VA64	Dissolution		223	0.113	190	0.151	194	0.159
	Desupersaturation	20			299	0.109	297	0.143
	Desupersaturation	40			222	0.117	260	0.124
	Dissolution				186	0.116	177	0.147

Seed-HPMC	Desupersaturation	0.5	146	0.196	380	0.219	323	0.142
	Dissolution				562	0.248	192	0.263
	Desupersaturation	5			318	0.114	252	0.112
	Dissolution				529	0.234	221	0.183
	Desupersaturation	20			283	0.213	326	0.230
	Desupersaturation	40			265	0.154	282	0.205
	Dissolution				544	0.241	197	0.114

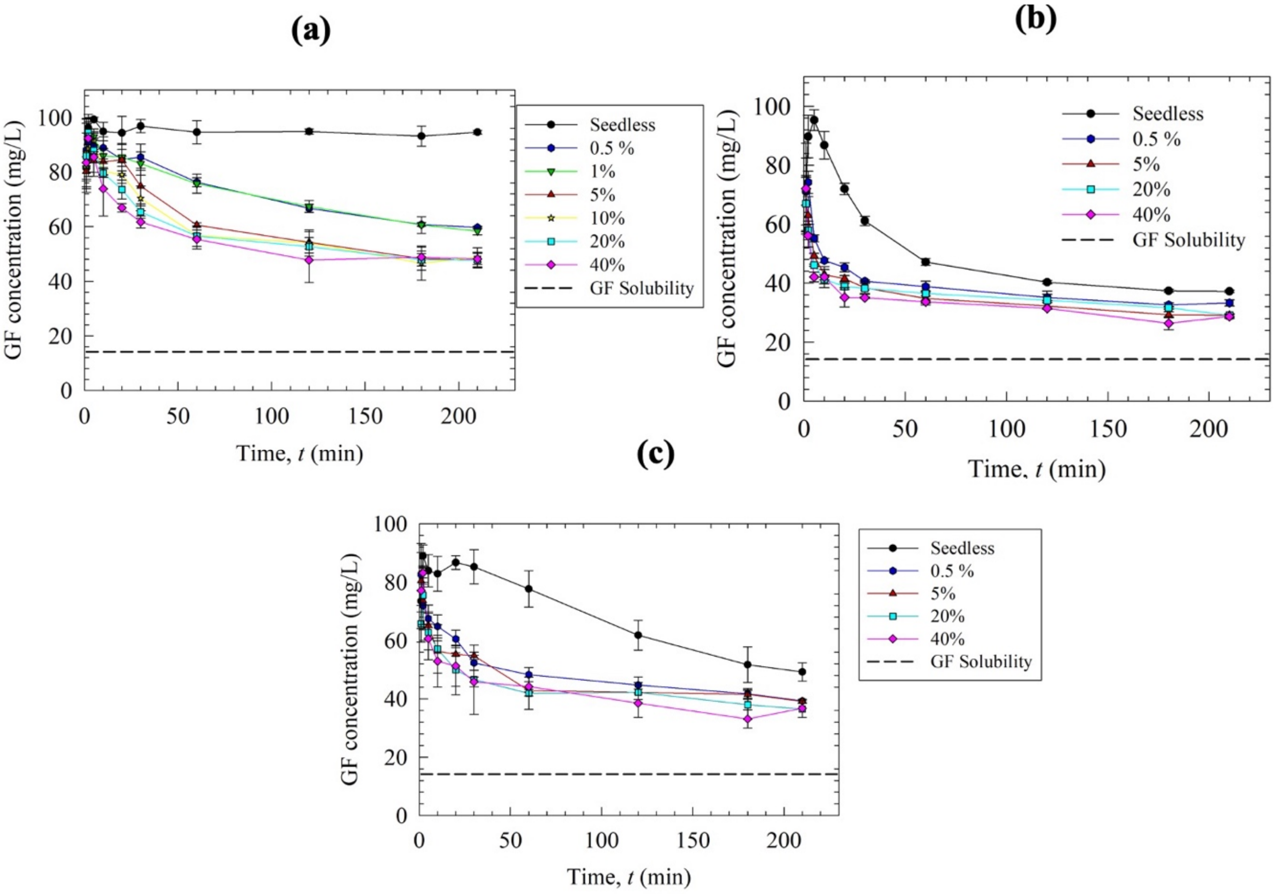

Figure S1. Effects of seed loading on the desupersaturation in the solvent-shift test when a 20 mL GF–acetone solution was mixed with 1000 mL aqueous solutions of SDS and various polymers: (a) Sol, (b) VA64, and (c) HPMC. Nanoseeds were added to the supersaturated solution at the weight percentages indicated.

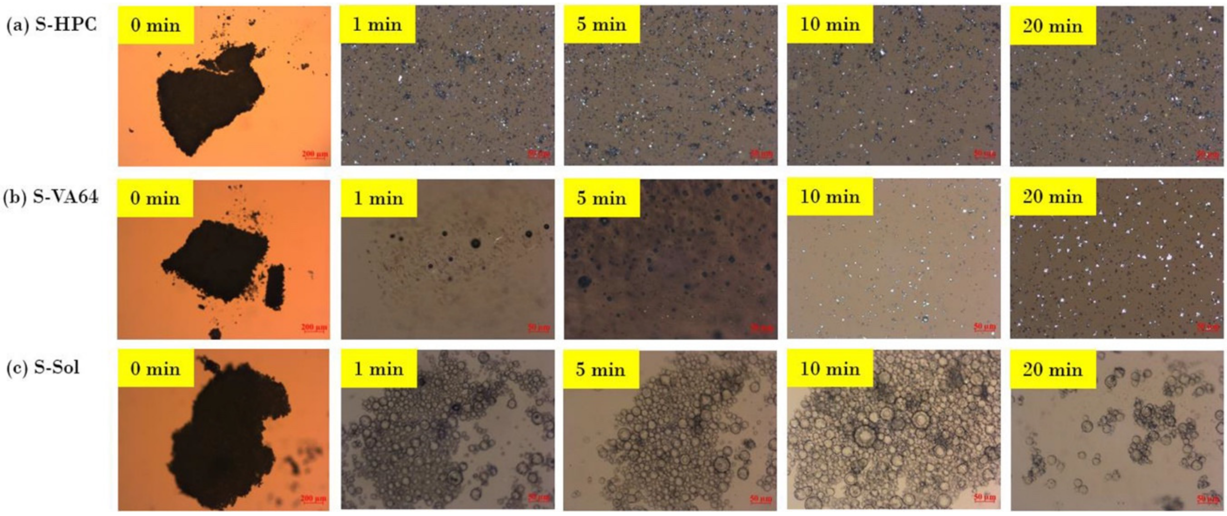

Figure S2. PLM images of a loose compact of the spray-dried ASD particles with 1:3 drug:polymer mass ratio in 40 μL deionized water: (a) S-HPC, (b) S-VA64, and (c) S-Sol, respectively. 20 μL deionized water was added initially and rest of the 20 μL water was added after 10 min. The images were taken at 0 (before adding water), 1, 5, 10, and 20 min after the addition of deionized water. Except 0 min image (5X magnification, scale bar: 200 μm), which focused on the compact, all other images focused on particles that emanated from the surface, which were captured at 20X magnification (scale bar: 50 μm). "Reprinted from *European Journal of Pharmaceutical Sciences* **2020**, *150*, 105354, Rahman, M.; Coelho, A.; Tarabokija, J.; Ahmad, S.; Radgman, K.; Bilgili, E., Synergistic and antagonistic effects of various amphiphilic polymer combinations in enhancing griseofulvin release from ternary amorphous solid dispersions Copyright (2022), with permission from Elsevier.
